# Luteolin-7-glucoside inhibits IL-22/STAT3 pathway, reducing proliferation, acanthosis, and inflammation in keratinocytes and in mouse psoriatic model

**DOI:** 10.1038/cddis.2016.201

**Published:** 2016-08-18

**Authors:** R Palombo, I Savini, L Avigliano, S Madonna, A Cavani, C Albanesi, A Mauriello, G Melino, A Terrinoni

**Affiliations:** 1 Department of Experimental Medicine and Surgery, University of Rome “Tor Vergata”, Via Montpellier, 1, Rome 00133, Italy; 2 Experimental Immunology Laboratory, Biochemistry Laboratory, IDI-IRCCS-FLMM, Via dei Monti di Creta, 104, Rome 00167, Italy; 3 Department Biomedicine and Prevention, University of Rome “Tor Vergata”, Via Montpellier, 1, Rome 00133, Italy; 4 Medical Research Council, Toxicology Unit, Hodgkin Building, Leicester University, Lancaster Road, P.O. Box 138, Leicester LE1 9HN, UK; 5 Biochemistry Laboratory, IDI-IRCCS-FLMM, Department of Experimental Medicine and Surgery, University of Rome “Tor Vergata”, Via Montpellier, 1, Rome 00133, Italy

## Abstract

The epidermis is a dynamic tissue in which keratinocytes proliferate in the basal layer and undergo a tightly controlled differentiation while moving into the suprabasal layers. The balance between keratinocyte proliferation, differentiation, and death is essential, and its perturbation can result in pathological changes. Some common skin diseases, such as psoriasis, are characterized by hyperproliferation accompanied by inflammatory reactions, suggesting that molecules with topical anti-inflammatory and ROS scavenging abilities may be useful for their treatment. Here we investigate the potential of the flavone Luteolin-7-glucoside (LUT-7G) as a treatment for psoriasis. We show that LUT-7G leads to a modification of the cell cycle and the induction of keratinocyte differentiation, with modification of energy, fatty acid, and redox metabolism. LUT-7G treatment also neutralizes the proliferative stimulus induced by the proinflammatory cytokines IL-22 and IL-6 in HEKn. Moreover, in the Imiquimod (IMQ) mouse model of psoriasis, topical administration of LUT-7G leads to a marked reduction of acanthosis and re-expression of epidermal differentiation markers. Dissection of the IL-22 signalling pathway, activated by IMQ treatment, demonstrates that LUT-7G impairs the nuclear translocation of phosphorylated (activated) STAT3, blocking the IL-22 signalling cascade. Thus LUT-7G appears to be a promising compound for the treatment of hyperproliferative and inflammatory skin diseases, such as psoriasis.

The epidermis is a dynamic tissue mainly constituted by keratinocytes that undergo a tightly controlled differentiation programme, moving from the basal to the suprabasal layers.^1^ Terminal differentiation is associated with several morphological, transcriptional, and metabolic changes of these cells. The outcome of this differentiation process is the conversion of keratinocytes into corneocytes, which form the stratum corneum.^2^ This special process of programmed cell death involves the complete breakdown of nuclear DNA and thereby terminates genetic control of cellular metabolism.^3, 4^ In the epidermal basal layer, cells express keratin 5 (KRT5) and KRT14, while in cells of the upper (spinous) layers these are progressively downregulated and replaced by KRT1 and KRT10, which reinforce cell–cell junctions and provide resistance against mechanical stress.^5, 6, 7^ A number of genes regulate epidermal development and differentiation, including transcription factors sucha as p63, a member of the p53 family,^8, 9, 10^ as well as microRNAs.^11, 12, 13^ Epidermal differentiation is paralleled by keratinocyte replicative senescence, another important process that prevents uncontrolled cell proliferation. Senescence is accompanied by growth arrest and changes in morphology and gene expression profile.^2, 14^

The balance between keratinocyte proliferation, differentiation, and replicative senescence is important to prevent pathological changes in the epidermis. Indeed epidermal homeostasis is altered in skin disorders, such as psoriasis, atopic dermatitis, and others, mainly characterized by an aberrant hyperproliferation of keratinocytes in the interfollicular epidermis, and by a significantly altered differentiation of keratinocytes. These changes are usually accompanied by inflammation.^15, 16^ Certain forms of ichthyosis are also characterized by hyperkeratinization and inflammation, as in hereditary genetic defects of transglutaminase 1, ABCA12, connexins, and keratins.^17, 18, 19^ Psoriatic and ichthyotic plaques have a scaly surface, which is caused by aberrant terminal differentiation of keratinocytes. The granular layer is greatly reduced or even absent in psoriatic lesions, with a stratum corneum formed by incompletely differentiated keratinocytes that aberrantly retain a cell nucleus. The scaling, and the resulting break in the protective barrier, are caused by the failure of psoriatic corneocytes to secrete extracellular lipids, to stack normally, and therefore to firmly adhere each other.^16^ Severe inflammation is also typical of psoriatic skin, and it is mostly driven by T lymphocytes, which locally release proinflammatory cytokines with the epidermis being the primary target.^15, 16^ Psoriatic keratinocytes also display resistance to cytokine-induced apoptosis.^20, 21^ In the skin, inflammation often also results in increased ROS levels. The intracellular redox state is a critical mediator of many metabolic, signalling, and transcriptional processes in cells, and an adequate balance between oxidizing and reducing conditions is essential for normal function and cell survival.^22, 23^ In pathological skin conditions, ROS levels are increased, and in particular, in psoriasis, oxidative stress is severely enhanced both systemically and locally in skin lesions.^24^ This suggests that molecules with topical anti-inflammatory and ROS scavenging abilities may be useful therapeutics in inflammatory skin diseases, such as psoriasis.

A particular class of natural molecules, having antioxidant and anti-inflammatory activity, are the polyphenols such as the flavonoids,^25, 26, 27, 28^ which are a class of secondary plant metabolites. They have a wide range of structures and are responsible for the major organoleptic characteristics of plant-derived foods and beverages, particularly colour and taste.^29^

Based on their molecular structure, flavonoids can be classified into six major subgroups: flavanols, flavanones, flavones, flavan-3-ols (catechins), anthocyanins, and isoflavones. Recently, interest in these substances has been stimulated by the potential health benefits arising from their antioxidant activity. The functional hydroxyl groups in flavonoids mediate their antioxidant effects by scavenging free radicals and/or by chelating metal ions. Among flavones, luteolin is a common dietary compound that is found in a large number of fruits and vegetables, including parsley, onions, wheat sprouts, chamomile, seasonings, tea, and orange.^30^ It is mainly present in a glycosylated form in plants, and the glycoside is hydrolysed during absorption.^31^ The hydroxyl group and 2−3 double bond represent an important structure in this compound, which is associated with its biochemical and biological properties, such as its antioxidant activity.

In this study, we investigate the ability of Luteolin-7-glucoside (LUT-7G) flavone in regulating proliferative responses, as well as differentiation in interleukin 22 (IL-22)-treated cultured human keratinocytes and in the imiquimod (IMQ)-induced (psoriasiform) model of psoriasis^32^ and show that this compound has important effects on these processes and could be a potential new drug for psoriasis.

## Results

### LUT-7G regulates senescence and differentiation in cultured human keratinocytes

We used the isomer, LUT-7-Glucoside (−7G), the major stable form of the flavone found in natural vegetables,^31^ to treat human normal keratinocytes (HEKn) *in vitro*. We initially performed experiments to evaluate the effects of LUT-7G on cell cycle and apoptosis. LUT-7G leads to an alteration of cell cycle progression with a consistent accumulation of cells in the G1 phase in a time- and dose-dependent manner (Supplementary Figures S1A and B). Under these experimental conditions, Lut-7G does not induce apoptosis in these cells (Supplementary Figure S1C). Interestingly Apigenin, a flavonoid closely related to luteolin but lacking a hydroxyl group in the 3′ position of the phenolic C-ring, showed no effect on the cell cycle of these cells while inducing apoptosis.

**Figure 1:**
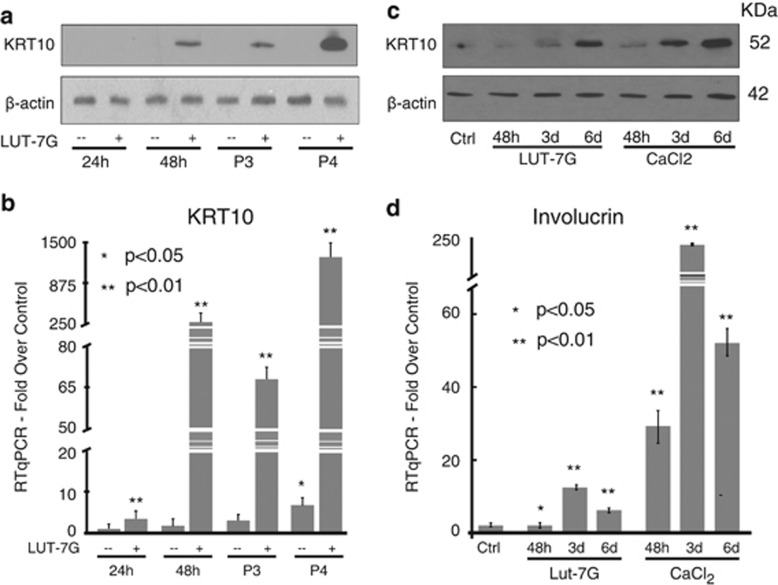
Luteolin-7G induces differentiation process. HEKn were maintained in culture from P2 to P4 with 20 *μ*M of LUT-7G. (**a**) Western blotting: The keratin transcript is visible upon 48 h of treatment with the flavone (P2), and it is strongly increased in P3 and P4 passages. (**b**) KRT10 qPCR analysis shows a strong transcriptional effect of the drug. (**c**) Cells at P3 were treated with the flavone and calcium, and the KRT10 protein levels have been analysed after 48 h, 3 and 6 for both treatment. (**d**) qPCR analysis of Involucrin expression demonstrates that both luteolin and calcium induce this differentiation marker at P3, with the same time course. Loading control=*β*-actin (**a** and **c**). Bars (**b** and **d**): ±S.D. *P<0.05; **P<0.01

We then investigated whether treatment with LUT-7G was affecting keratinocyte differentiation by looking at KRT10 expression. As shown in Figures 1a and b, cells cultured in LUT-7G-containing medium from P2 to P4 passages showed an increase of KRT10 both at the protein and mRNA levels. The morphological analysis of LUT-7G-treated keratinocytes by confocal microscopy confirmed the increase of KRT10 expression in keratinocyte cytoskeleton (Supplementary Figures S1D and E), again indicating the beginning of the differentiation. Time course experiments performed using P3 cells, treated with LUT-7G for 48 h, 3 and 6 days, confirmed that the flavone upregulates KRT10 protein levels in a time-dependent manner (Figure 1c, lanes 1–4).

This prodifferentiative action of LUT-7G was comparable with that obtained in cells cultured in 1.2 mM calcium-containing medium, which is well known to promote keratinocyte differentiation ‘*in vitro*'^33, 34^ (Figure 1c). In the same cell system, we also analysed the expression of another epithelial differentiation marker, involucrin, which is specifically expressed in the suprabasal layers of stratified squamous epithelia.^35^ Again, LUT-7G induced expression of involucrin transcripts (Figure 1d), although with different kinetics to that obtained with calcium treatment.

### Metabolism, lipid synthesis, redox state, and inflammatory responses are reduced in keratinocytes treated with luteolin

During the differentiation programme, keratinocytes are transformed into corneocytes through major modifications of the cell structure and function, with loss of the main intracellular structures, such as nucleus, RE, and Golgi.^1^ Furthermore, during epidermal development and differentiation, there are some important changes in lipid synthesis to accomplish the required modifications of the epidermal barrier. Keratinocytes require fatty acids as substrates for the formation of phospholipids and sphingolipids, which are key components of the lipid-enriched lamellar bodies. These organelles secrete their lipid content (glucosylceramides, sphingomyelin, phospholipids, and cholesterol) and lipid-processing enzymes, which make the upper layers waterproof.^36^ In addition, the epidermis needs ceramides, essential for providing anchors for the membrane system to the external surface of the corneocyte scaffold. Indeed, epidermal lipids comprise considerable quantities of ceramides that contain very long-chain *N*-acylated fatty acids (C24–C30).^37^ For this reason, we performed a metabolomics analysis using P3-passaged HEKn keratinocytes treated for 3 days with LUT-7G.

**Figure 2:**
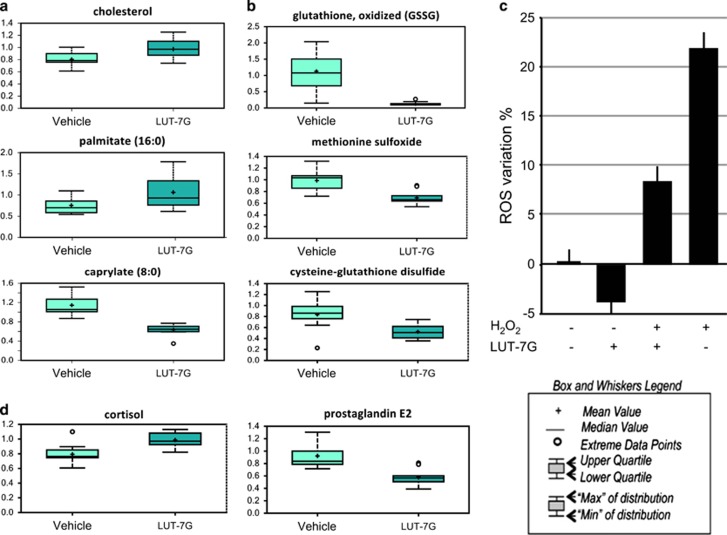
Lipids, redox, and inflammatory molecule analysis. (**a**) The modification of lipid expression pattern during Lut-7G treatment is evidentiated by the increase of cholesterol, palmitate, and the concomitant reduction of caprylate. (**b**) Effect of LUT-7G (20 *μ*M) showed a decreased levels of antioxidant metabolites such as glutathione in its oxidized form, methionine sulfoxide, and cysteine-glutathione disulfide. Intracellular ROS detection (24 h) in HEKn control, H_2_O_2_-, and LUT-7G-treated cells. (**c**) The LUT-7G treatment display an antioxidative effect. (**d**) Cells treated with luteolin show higher level of cortisol and lower level of prostaglandin E2, important metabolites in the inflammation processes

The results of this analysis shows higher cholesterol levels in luteolin-treated cells, demonstrating the promotion of lipid raft generation (Figure 2a, upper panel).^38^ Indeed, long-chain fatty acids such as palmitoleate, stearate, and linoleate were elevated in luteolin-treated samples, while diminished levels of medium-chain fatty acids such as caproate and caprylate were found in luteolin-treated cells and may reflect decreased fatty acid *β*-oxidation (Figure 2a, lower panels, one for each class) and resembles natural keratinocyte differentiation.^38^ Metabolomic analysis of LUT-7G-treated cells also show some important changes in molecules involved in the redox scavenging pathways. In fact, in LUT-7G treated cells, GSSG, methionine sulfoxide, and CYSSG levels are reduced (Figure 2b). These results support the idea that LUT-7G itself can act as a scavenger, reducing the oxidation of GSH. This effect has been confirmed by evaluating the content of oxygen reactive species by cytofluorimetric analysis (Figure 2c). The redox equilibrium is strictly linked with the inflammation processes, and consistently, the analysis of this pathway showed that LUT-7G increases the levels of the anti-inflammatory, cortisol, accompanied by a decrease in levels of the proinflammatory prostaglandin E2 (Figure 2d), strongly suggesting that LUT-7G can have important anti-inflammatory effects in keratinocytes.

### LUT counteracts the proliferative effects of IL-22 in keratinocytes and reduces the psoriasiform phenotype in IMQ-treated mice

**Figure 3:**
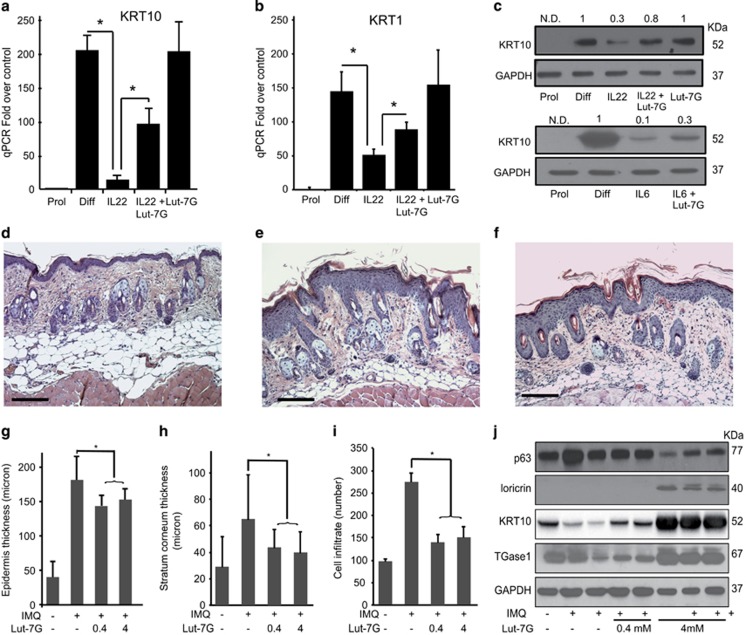
Luteolin-7G treatment counteracts effects of IL-22 pathway. P3 confluent cells have been treated for 4 days with IL-22 and LUT-7G. (**a**) KRT1 and (**b**) KRT10 qRT-PCR analysis shows reduction of the IL-22-induced effects in cells co-treated with LUT-7G (*P<0.01, test-*T*, two tails). (**c**) Western blotting analysis of KRT10 expression after treatment with IL-22 (upper panel) and IL-6 (lower panel), demonstrating reversion of expression by treatment with LUT-7G. Densitometric analysis represented as fold over control above each panel. Representative H&E staining of (**d**) untreated, (**e**) treated with IMQ cream, and (**f**) in the presence of LUT-7G (4 mM) mice skin sections. After IMQ treatment, the typical thickening of the epidermis is visible, reduced by LUT-7G addition. (Bars: 200 *μ*m). Mice skin treated with IMQ reverted their condition after LUT-7G topical application of 0.4 and 4 mM. The quantification of (**g**) epidermal, (**h**) scale thickness, and (**i**) cell infiltrate number were analysed as parameters of skin acanthosis and inflammation. Plots show means of microns of epidermis and stratum corneum thickness, and mean of number of cells infiltrating dermis per section, ±S.D. per group (*n*=4–6 mice, *P<0.001). Western blotting shows p63, loricrin, KRT10, and TGase1 modifications upon treatment with LUT-7G (**j**)

IL-22 is a key pathogenic cytokine in psoriasis, as it can trigger regenerative and proliferative programmes in keratinocytes, inhibit their differentiation, and activate the inflammatory cascade, inducing proinflammatory molecules.^39^ It is released by T helper type 17 (Th17) and Th22 subsets, mainly targets keratinocytes in the skin, and triggers STAT3-dependent pathways.^39^ We evaluated whether LUT-7G could antagonize IL-22 signalling cascade activation in keratinocytes and restore the differentiated phenotype. Primary cultures of keratinocytes undergoing differentiation (P3, 4 days of growth after confluence) express high levels of the two cytokeratins KRT1 and KRT10 (Figures 3a and b), which are essentially abrogated by IL-22, as previously described.^39^ In these keratinocyte cultures, LUT-7G administrated together with the cytokine reverts the IL-22-induced effect, restoring KRT1 and KRT10 expression (Figures 3a and b, WB in Figure 3c, upper panel). To confirm the ability of the flavone to counteract the proliferative effects promoted by chemokines, we performed a treatment also with IL-6. This chemokine is able to trigger the same signalling cascade activated by IL-22, stimulating keratinocytes to proliferate. Again, treatment with LUT-7G counteracts the effect of IL-6 on KRT10 expression and on differentiation (Figure 3c).

We then evaluated the effects of LUT-7G treatment ‘*in vivo*' in a murine model of skin inflammation. We used the IMQ-induced psoriasiform mice model, which closely resembles the human psoriatic lesions in terms of phenotypic and histological characteristics, including epidermal hyperplasia (acanthosis) and the presence of inflammatory infiltrates in the dermis.^32, 40^ We topically treated the dorsal skin of mice repeatedly with IMQ for 5 days to induce a psoriatic-like skin inflammatory responses. LUT-7G was administered together with IMQ for 5 days, at different concentrations (0.4 and 4 mM), and the skin of the treated mice was then analysed by histology and immunohistochemistry. As evident in H&E-stained skin sections, 5 days of IMQ treatment in mice result in scaling and parakeratosis of the stratum corneum and epidermal acanthosis and widespread inflammatory infiltrates, as compared with controls (Figures 3d and e). In particular, IMQ strongly induced thickening of both epidermis and stratum corneum, as assessed by quantifying the average of epidermal and scale thickness on images of skin sections (Figures 3g–i). As expected, IMQ treatment also results in epidermal acanthosis in mouse skin by inducing hyperproliferation and reducing the differentiative process. These parameters were evaluated analysing keratinocyte proliferation markers, such as p63, and differentiation ones such as KRT10, TGase1, or loricrin, in protein lysates from mouse skin biopsies (Figure 3j).

When IMQ was co-administered with LUT-7G, its effects on the mouse skin were significantly reduced, in fact, both epidermal and scale thickness decreased in response to LUT-7G treatment (Figures 3g–i). Moreover LUT-7G treatment results in a dose-dependent reduction of the keratinocyte proliferation marker p63 and the increase of the differentiation markers Loricrin, TGase1, and KRT10. Indeed, a dose–response effect was observable evaluating molecular markers trends from skin extracts of treated mice. In this experiment, the keratinocyte proliferation marker p63 is reduced by LUT-7G; conversely, markers of differentiation such as Loricrin, TGase1 and KRT10 are restored by the treatment with the drug (Figure 3j).

**Figure 4:**
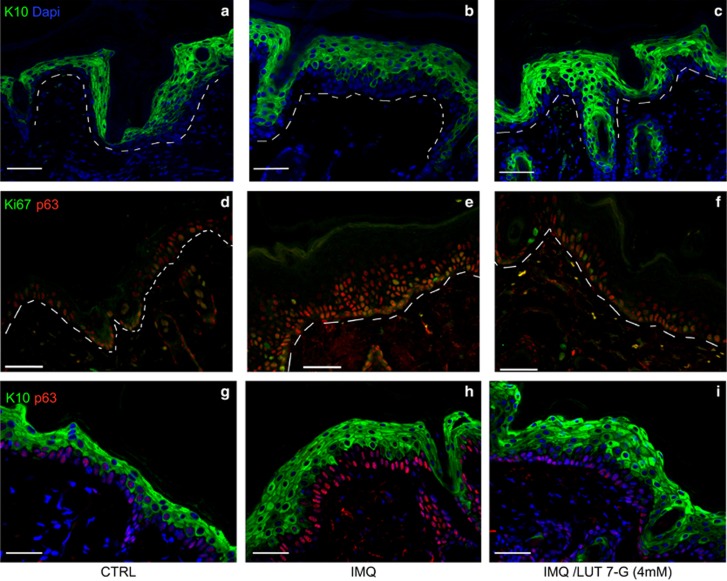
Luteolin-7G counteracts IMQ effects in the mice skin. Confocal immunofluorescence analysis show (**a**) reduction of positive KRT10 cell layer numbers after LUT-7G treatment if compared with (**b**) IMQ and (**c**) normal control. Ki67 and p63 immunostaining demonstrate that (**f** and **i**) LUT-7G-treated skin has a proliferation rate similar to the (**d** and **g**) untreated; conversely, (**e** and **h**) IMQ-treated skin shows a higher number of proliferating cell layers with respect to controls (**d** and **g**). Dotted line divides epidermis (top) and dermis (bottom). Bars: 50 *μ*m

To deeply analyse the modification of epidermal structure and cell proliferation using LUT-7G in IMQ mouse model, we used a confocal analysis to dissect the expression of specific proteins in epidermal layers. The results show as expected the increase of basal and granular layer (KRT10 positive) in the hyperproliferative mice skin when treated with IMQ (Figure 4b) while sections of mice treated with both IMQ and LUT-7G appear more similar to the untreated mice showing a reduction of the granular layer, with increase of KRT10 expression compared with the IMQ-only-treated mice (Figures 4a–c). We also evaluated the proliferation state of mice epidermal cells by staining for p63 and Ki67, and show that concomitant treatment with LUT-7G leads to the reduction of layers positive for both Ki67 and p63 observed in IMQ-treated animals, almost restoring the situation observed in normal untreated skin (Figures 4d–i).

Thus LUT-7G appears to revert the induction of the psoriasiform phenotype, including proliferative and inflammatory responses in the IMQ mouse model in a dose-dependent manner.

### Luteolin-7G blocks the nuclear translocation of STAT3

In order to identify the mechanism of action of LUT-7G, we analysed its effect on the signalling pathway activated by IL-22. It is known that IL-22 binds to a heterodimeric receptor complex (IL-22R1 and IL-10R2), and signal transduction occurs via JAK protein activation that in turn determines STAT3 phosphorylation.^41^ Phosphorylation in serine 727 (p-S727) has been described, but only p-Y705 is absolutely required for STAT3-mediated self-renewal in epithelial stem cells and proliferation.^42^ STAT3 activation mediated by its phosphorylation is strictly dependent on the acetylation of its Lys 685 residue, a posttranslational modification occurring through p300 acetylase and counteracted by histone deacetylase SIRT1.^43, 44^

Activated (phosphorylated) STAT3 is translocated from cytoplasm to the nucleus, where it induces the transcription of downstream genes that are involved in proliferation and cell cycle regulation. One of the best known effects, moreover in psoriatic pathway, is the induction of acanthosis (keratinocyte proliferation) and dermal inflammation.^45^

**Figure 5:**
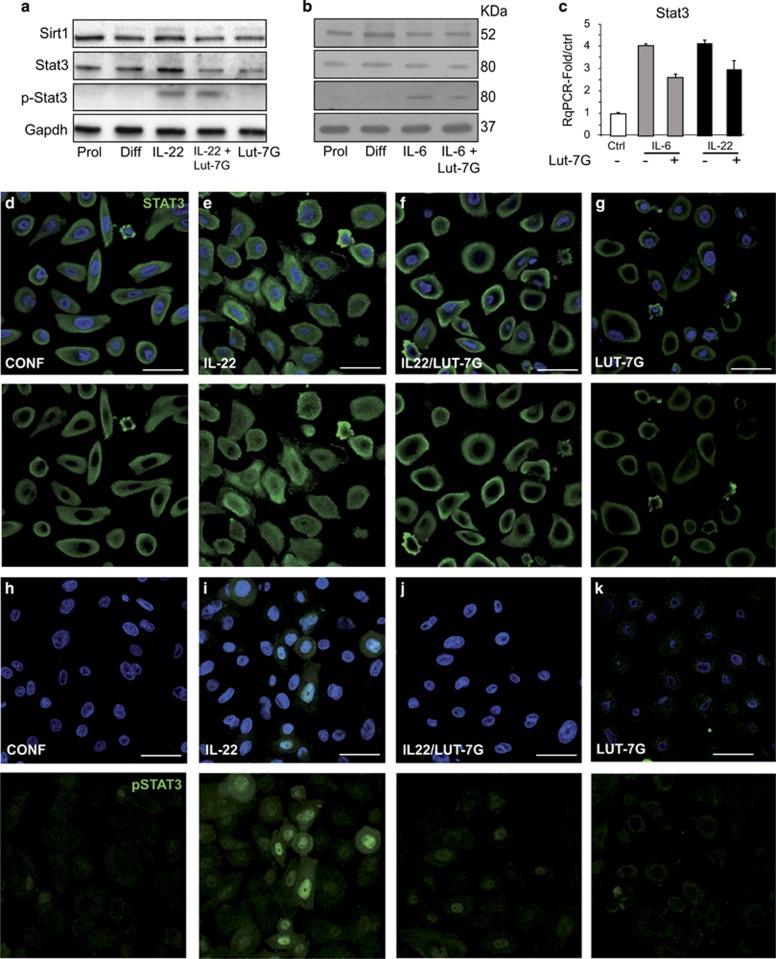
STAT3 nuclear translocation is blocked by Luteolin-7G. (**a** and **b**) HEKn protein extract from IL-22 and IL-6 treatments were analysed for protein expression levels of SIRT1 and STAT3 (total and phosphorylated) by western blotting and (**c**) transcriptionally by RT-qPCR. (**a** and **b**) STAT3 activation (phosphorylation on Tyr 705) is enhanced by IL-22 treatment and LUT-7G do not revert this process. Confocal microscopic investigation show a clear nuclear localization of STAT3 in (**e**) IL-22-treated cells with respect to (**d**) the control cells treated only with LUT-7G, (**f**) the addition to the culture medium of LUT-7G revert the nuclear staining for STAT3. The analysis of HEKn cells treated with IL-22 and LUT-7G with anti-Phospho-STAT3 shows the nuclearization of pSTAT3 upon (**i**) IL-22 treatment with respect to the (**h**) control and (**j**) the reversion of this effect after LUT-7G treatment. (**k**) cells treated only with LUT-7G

As shown in Figures 5a–c, Lut 7G had no effect on STAT3 or SIRT1 steady-state levels nor on STAT3 phosphorylation induced by IL-22 or IL-6 treatment. Interestingly, however, it appeared to block IL-22 translocation to the nucleus (Figures 5d–k) We investigated the modulation of STAT3 in HEKn treated with IL-22 and LUT-7G, comparing also with IL-6 treatment (Figures 5a–c), known to activate the same pathway.^46^ In both cases, the levels of both STAT3 transcript and protein is only slightly modified by the two cytokines. In addition, SIRT1 levels are also not evidently modified, indicating that its de-acetylation reaction on STAT3 is not regulated by the LUT-7G treatment (Figures 5a and b), demonstrating that it is not involved in the inhibition of the pathway. As the active form of STAT3 is the p-Y705, we analysed whether LUT-7G was able to modify the phosphorylation state induced by IL-22 and IL-6. As visible from the WB in Figures 5a and b, there is no effect in this modification upon treatment, indicating that LUT-7G treatment is not able to revert or block STAT3 phosphorylation in Y705 residue, and it does not modify the steady-state level of Stat and Sirt1 (Figure 5a, last lane). To activate the transcription of downstream genes, the phosphorylated protein should be translocated to the nucleus; thus we investigated, using confocal immunofluorescence analysis, the subcellular localization of STAT3 under different conditions. The results show that, in confluent (control) cells, STAT3 is not phosphorylated (Figure 5a, lane 2) and with a cytoplasmic localization (Figure 5d). During IL-22 treatment, part of STAT3 is phosphorylated according to WB analysis (Figure 5a, lane 3), and it is translocated into the nucleus (Figure 5e). Importantly, combined treatment with IL-22 and LUT-7G also does not abolish STAT3 phosphorylation on tyrosine 705 (Figure 5a, lane 4) and seems to impair its translocation to the nucleus (Figure 5f). To verify the nuclear translocation impairment, exerted by LUT-7G treatment, we used the p-Y705-STAT3 antibody. The results showed that the p-Y705-STAT3 antibody staining is visible only in the nucleus of IL-22-treated cells (Figure 5h), and the signals strongly decrease, after LUT-7G treatment (Figure 5i), confirming the effect of LUT-7G in blocking the nuclear translocation of the transcription factor. Even in this case, LUT-7G treatment modulated the expression of STAT3 and its posttransductional modifications, similarly to that obtained in the differentiated condition, and it does not modify the subcellular localization of Stat3 when used alone in confluent cells (Figures 5d and k).

**Figure 6:**
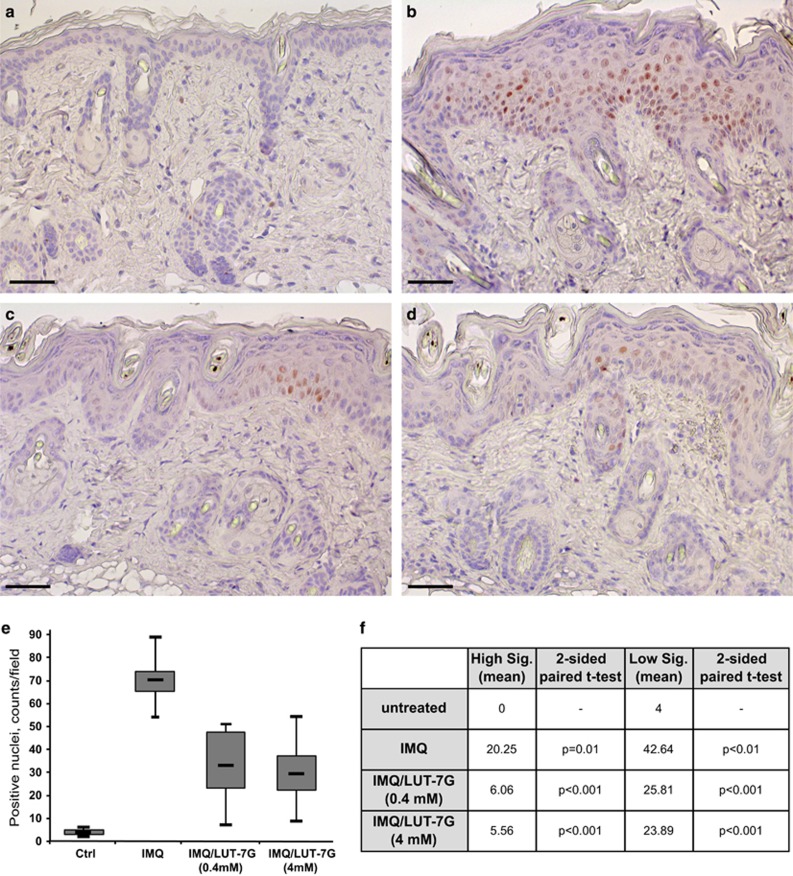
Immunohistochemical analysis of phosphorylated STAT3 in the IMQ mice skin. The reduction in the pSTAT3 staining is obtained by ‘*in vivo*' treating IMQ mice skin with LUT-7G: in control section, (**a**) pSTAT3 is almost absent, (**b**) while it is evident in IMQ mice tissue sections. Treatment with LUT-7G reduces dramatically the presence of pSTAT3 in keratinocyte nuclei at (**c**) 0.4 mM and (**d**) 4 mM concentration. Statistical analysis performed by counting the total number of stained positive nuclei (**e**) shows a strong reduction of IMQ effect. (**f**) The differences are significant also when calculated between two class of positive nuclei: low and high signal. (Bars: 35 *μ*m)

We confirmed the effect of LUT-7G on p-STAT3 translocation to the nucleus *in vivo* in the mice model described before. Indeed, in control mouse tissue, the staining for p-STAT3 is absent (Figure 6a), but it becomes evident and with a clear nuclear localization after IMQ treatment (Figure 6b). LUT-7G treatment strongly reduces the presence of p-STAT3 in the nucleus of treated mouse skin in a dose-dependent manner (Figure 6c, 0.4 mM and Figure 6d, 4 mM). In mice treated with LUT-7G (Figure 6e), the statistical analysis, resulted by the counting of positive stained nuclei, showed a significative reduction of above the 55% of p-STAT3 protein, even when the positive nuclei are distinguished between high and low signal (Figure 6f). No evident changes are obtained by treating the mice skin only with LUT-7G (Supplementary Figure S2).

In conclusion, these experiments confirm the ability of LUT-7G to counteract the proliferative and inflammatory stimuli used to induce a psoriatic phenotype, ‘*in vitro*' with the IL-22/IL-6 system and ‘*in vivo*' in the IMQ mouse model. We demonstrated that LUT-7G treatment exerts these effects by blocking the nuclear translocation of phosphorylated STAT3 and counteracting the transcriptional cascade activated by this transcription factor.

## Discussion

Current therapies for psoriasis and psoriatic arthropathy include corticosteroids and monoclonal antibodies and other antagonists to TNF*α*, although antibodies and other reagents directed towards other cytokines and immune receptors are under development (reviewed in Feely *et al.*^47, 48^). However, none of these agents are entirely satisfactory, giving rise to several side effects in many patients, particularly when administered over long periods of time. There is therefore need for novel therapeutic approaches.

Psoriasis is associated with activation of an IL-22 and IL-6/STAT3 pathway,^49, 50^ which contributes to keratinocyte hyperproliferation and failure to exit the cell cycle with the inability to fully mature into corneocytes. Indeed STAT3 could represent a good therapeutic target.^51^ Here we show that LUT-7G can block this pathway both *in vitro* and *in vivo*. These results were confirmed in the IMQ ‘*in vivo*' murine psoriasis model, in which the hyperproliferation and inflammation were IL-22/STAT3 dependent.^40^ In fact, the concurrent treatment with LUT-7G led to reduced expression of proliferation markers, increased production of markers of differentiation, and to a phenotypic improvement. Thus LUT-7G strongly reverted the inflammation and the cell proliferation induced by IMQ. To confirm the results, we also used IL-6 to induce keratinocyte proliferation and activation of the transcription factor STAT3.

In this paper, we report novel findings about the use of a natural flavonoid, LUT-7G, with beneficial pharmacological properties on hyperproliferative and inflammatory skin conditions. We show that the administration of LUT-7G, when used at micromolar concentrations, is able to modify the cell cycle ‘*in vitro*' with a corresponding switch from proliferation to differentiation. Interestingly, Apigenin, a flavonoid closely related to luteolin but lacking a hydroxyl group in the 3′ position of the phenolic C-ring, had no effect on differentiation (Supplementary Figure S1), reinforcing the idea that small molecular changes can result in profound changes in biological activity.

LUT-7G is known to reduce production of proinflammatory PGE2 by activated macrophages^52^ and has been proposed to have antiasthmatic activity both by reducing PGE2 and Th2 activity^53^ and to promote wound healing in the skin.^54^ In the present study, we confirm the inhibitory effect of LUT-7G on PGE2 but demonstrate that its anti-inflammatory effects are also due to increased production of cortisol. Most importantly, we show that *in vivo* LUT-7G is capable of reverting the inflammatory and cell proliferative phenotype induced by IMQ, suggesting that it could be a potential treatment for psoriasis. Our data suggest that LUT-7G blocks the signalling of IL-22 pathway by impairing the translocation of phosphorylated (activated) STAT3 from the cytoplasm to the nucleus (Supplementary Figure S3) independently from its phosphorylation.

In conclusion, the ‘*in vitro*' and ‘*in vivo*' data presented in this paper strongly suggest that the topical use of the natural product, LUT-7G, may be considered as possible treatment for psoriasis with reduced side effects.

## Materials and Methods

### Cell culture and treatments

HEKn (Cascade by Invitrogen, Carlsbad, CA, USA) were cultured in Epilife medium with human keratinocyte growth supplements added (Cascade). Cells were seeded on collagen-coated dishes and kept constantly subconfluent to avoid triggering of differentiation. At each passage, cells were harvested, counted, and collected to extract RNA and proteins. Cells were induced to differentiate by adding 1.2 mM CaCl_2_ to the culture medium for different time periods (2, 3, or 6 days). Otherwise, terminal differentiation of keratinocyte cultures was achieved by growing cells at 100% of confluence and thus keeping them in culture for another 4 days.

Apigenin 7-glucoside and LUT-7G (Sigma Aldrich, Milan, Italy) were dissolved in DMSO and stored at +4 °C kept protected from light. DMSO was used at a final concentration of 0.01% v/v in media, whereas IL-22 and IL-6 (R&D Systems, Minneapolis, MN, USA) were added to keratinocyte cultures at a final concentration of 50 ng/ml.

### IMQ model of psoriasiform-like skin inflammation

C57BL/6 mice (Harlan Laboratories, San Pietro al Natisone, Italy) were employed in all the experiments. Shaved mouse dorsal skin was treated daily for 5 consecutive days with 50 mg Aldara cream containing 5% IMQ (Meda AB, Solna, Sweden). On day 5, full-thickness skin biopsies of the treated area were collected with a 8-mm biopsy puncher. Skin was either snap frozen in liquid N_2_ for protein lysates preparation, or fixed in neutral buffered formalin (Sigma-Aldrich, St. Louis, MO, USA) for histopathological analysis. In some experiments, 50 *μ*l Aldara cream was mixed with LUT-7G (in DMSO solution) at a final concentration of 4 and 0.4 mM. Average epidermal and scale thickness was quantified by a researcher blind to the experimental groups who took five measurements per three sections for each mouse. Cells infiltrating dermis were also counted in three skin sections for each mouse.

### Cell viability and cell cycle analysis

Cells were harvested using 0.25% trypsin, washed with phosphate-buffered saline (PBS), and fixed with a 1 : 1 PBS and methanol–acetone (4 : 1 (v/v)) solution at −20 °C, treated with 50 *μ*l of a solution of 13 Kunitz/ml RNaseA at 37 °C for 15 min, and then stained with 50 mg/l propidium iodide (Sigma) for 30 min. Cell cycle was analysed using a FACS Calibur flow cytometer (BD Biosciences, New Jersey, NJ, USA) and 10 000 events were evaluated using the Cell Quest (BD) software.

### RNA extraction and quantitative real-time RT-PCR

MirVana miRNA Isolation Kit (Ambion by Invitrogen) was used for total RNA extraction. In all, 1000 ng of total RNA were reverse transcribed using the GoScript Reverse Transcription System (Promega, Madison, WI, USA), whereas real-time PCR was performed using GoTaq qPCR Master Mix (Promega). The expression of each gene was defined from the threshold cycle (Ct), and relative expression levels were calculated by using the 2^−(ΔΔCt)^ method. Primer pairs used in PCR reactions are listed in the table reported in Supplementary Table S1. Statistical analyses were performed using the unpaired Student's *t*-test, calculated on ΔCt.

### Western blotting

Protein lysates form cell cultures or skin biopsies were resolved on SDS polyacrylamide gels and blotted onto a Hybond P PVDF membrane (G&E Healthcare, Waukesha, WI, USA).

The following antibodies were used: anti-*β*-actin (Sigma; 1 : 5000), anti-GADPH (Sigma Aldrich; 1 : 5000), anti-K10 (Covante Inc., Princeton, NJ, USA; 1 : 1000), anti-TGase1 (Santa Cruz Biotechnology, Santa Cruz, CA, USA; 1 : 300), anti-loricrin (Covance; 1 : 1000), anti-p63 (Y4A3 p3362; Sigma-Aldrich; 1 : 1000), anti-phospho-STAT3 (Tyr 705) (Cell Signaling, Danvers, MA, USA; 1 : 1000) anti-Stat3 (C-20) (Santa Cruz; 1 : 100), and anti-SIRT1 (ab13749) (Abcam; 1 : 200).

### Metabolomic analysis

Cell were harvested at passage 3 (P3) after 3 days of treatment. Control cells were growth with a final concentration in medium of 0.01% of DMSO v/v (vehicle used to dissolve the flavone), and treated cells were growth with the addition of LUT-7G 20 *μ*M in medium. For each condition, 9 replicates were obtained, with 10 × 10^6^ cells each.

Samples were immediately stored at −80 °C and, at the time of analysis, were extracted and prepared for analysis using a standard metabolic solvent extraction method. Briefly, the extracted samples were split into equal parts for analysis by gas chromatography/mass spectrometry (GC/MS) or liquid chromatography/mass spectrometry (LC/MS/MS) platforms. Also included were several technical replicate samples created from a homogeneous pool containing a small amount of all study samples. The LC/MS portion of the platform was based on a Waters ACQUITY UPLC (Milford, MA, USA) and a Thermo-Finnigan LTQ mass spectrometer (San Jose, CA, USA), which consisted of an electrospray ionization source and linear ion-trap mass analyser. The sample extract was split into two aliquots, dried, and then reconstituted in acidic or basic LC-compatible solvents, each of which contained ≥11 injection standards at fixed concentrations. One aliquot was analysed using acidic positive ion optimized conditions and the other using basic negative ion optimized conditions in two independent injections using separate dedicated columns. Extracts reconstituted in acidic conditions were gradient eluted using water and methanol, both containing 0.1% formic acid, while the basic extracts, which also used water/methanol, contained 6.5 mM ammonium bicarbonate. The MS analysis alternated between MS and data-dependent MS2 scans using dynamic exclusion. The samples destined for GC/MS analysis were re-dried under vacuum desiccation for a minimum of 24 h prior to being derivatized under dried nitrogen using bistrimethyl-silyl-triflouroacetamide. The GC column was 5% phenyl and the temperature ramp is from 40 °C to 300 °C in a 16 min period. Samples were analysed on a Thermo-Finnigan Trace DSQ fast-scanning single-quadrupole mass spectrometer using electron impact ionization. The instrument was tuned and calibrated for mass resolution and mass accuracy on a daily basis. The information output from the raw data files was automatically extracted.

For ions with counts >2 million, an accurate mass measurement could be performed. Accurate mass measurements could be made on the parent ion as well as fragments. The typical mass error was <5 p.p.m. Ions with less than two million counts require a greater amount of effort to characterize. Fragmentation spectra (MS/MS) were typically generated in a data-dependent manner, but if necessary, targeted MS/MS could be employed, such as in the case of lower-level signals. Compounds were identified by comparison with library entries of purified standards or recurrent unknown entities. Identification of known chemical entities was based on comparison with metabolic library entries of purified standards. The combination of chromatographic properties and mass spectra gave an indication of a match to the specific compound or an isobaric entity.

Instrument variability was determined by calculating the median relative standard deviation (RSD) for the internal standards that were added to each sample prior to injection into the mass spectrometers. Overall process variability was determined by calculating the median RSD for all endogenous metabolites (i.e., non-instrument standards) present in 100% of the matrix samples, which are technical replicates of pooled client samples. The metabolic analysis comprises a total of 279 compounds named biochemicals. Following imputation of any missing values present with the minimum observed value for each compound, normalization to Bradford protein concentration, and log transformation of median scaled data, Welch's two-sample *t*-test was used to identify biochemicals that differed significantly between experimental groups. A summary of the numbers of biochemicals that achieved statistical significance (*P*≤0.05), as well as those approaching significance (0.05<*P*<0.10), is shown below.

An estimate of the false discovery rate (*q*-value) is calculated to take into account the multiple comparisons that normally occur in metabolomic-based studies. For example, when analysing 200 compounds, we would expect to see about 10 compounds meeting the *P*≤0.05 cutoff by random chance. The *q*-value describes the false discovery rate; a low *q*-value (*q*<0.10) is an indication of high confidence in a result. Although a higher *q*-value indicates diminished confidence, it does not necessarily rule out the significance of a result.

### ATP level detection

After 3 days, treatment cells were harvested, counted, and analysed by bioluminescence. ATP was measured based on luciferin–luciferase reaction using the ADP/ATP Ratio Assay Kit (Abcam) as per the manufacturer's instructions.

### ROS detection

Oxidative stress was induced by 100 *μ*M H_2_O_2_ addition to culture medium. Keratinocytes were collected and analysed after treatment. To detect ROS levels, a chloromethyl derivative of H_2_DCFDA (CM-H_2_DCFDA, Invitrogen), an indicator for reactive oxygen species, was used. This dye is a non-polar compound that diffuses into cells. Cells were collected and washed once with 1% PBS. Then they were incubated for 30 min at 37 °C in PBS containing CM-H_2_DCFDA (final concentration 10 *μ*M). DCF fluorescence was detected by FacsCalibur flow cytometer (Becton Dickinson, BD Biosciences) using excitation at 485 nm and emission at 520 nm.

### Immunofluorescence and skin histopathology

After treatment, cells were formalin fixed at room temperature for 10–15 min, permeabilized with 0.5% Triton X-100 in PBS for 10 min, washed three times for 10 min in PBS, and incubated for 1 h in blocking buffer. Fixed skin was embedded in paraffin and tissue sections were deparaffinized and stained with H&E for histological analysis. Antigen retrieval was achieved by microwaving sections in 0.01 M sodium citrate (pH 6). After an overnight incubation in Sodium Borohydride (Sigma), the sections were blocked with 5% goat serum in 1 × PBS for 2 h. Tissue sections and cell slides were incubated with the primary antibodies. The immunofluorescence staining were performed using anti-p63 (clone 4YA3, Sigma, 1/400 dilution), anti-Ki67 (Novocastra, Newcastle upon Tyne, UK; 1 : 1300), anti-K10 (Covance; dilution 1 : 1000), anti phospho-STAT3 (Tyr 705) (D3A7) XP (Cell Signaling; 1 : 200), and anti-Stat3 (C-20) (Santa Cruz; 1 : 100). The following secondary antibodies were used to develop immunoreactivities: Alexa Fluor 488 goat anti-rabbit IgG (H+L) antibody and Alexa Fluor 568 goat anti-mouse IgG (H+L) antibody (both from Invitrogen). Nuclei were stained with DAPI. Cells and tissue sections were mounted using the Prolong Antifade Kit (Invitrogen). For immunohistochemical analyses, sections were deparaffinized, washed by BioClear (Bio-Optica, Milano, Italy), and rehydrated in solutions with concentrations decreasing alcohol and increasing water. H&E images were acquired at × 10 magnification with an Olympus VS120 slide scanner (Olympus Italia Srl, Milano, Italy). Average epidermal and scale thickness was quantified by a researcher blind to the experimental groups who took five measurements per three sections for each mouse. Otherwise, slides were analysed with a confocal laser microscope (NIKON Eclipse Ti, Nikon-Italia, Firenze, Italy). Detection of the signal was performed using the NIS element AR4.00.04 software (Nikon, Nikon-Italia).

### Statistical analysis

For ‘*in vivo*' experiments, the significance of differences between experimental groups (mice treated with IMQ *vs* mice treated with IMQ plus LUT-7G 100X or LUT-7G 1000X) were calculated by unpaired Student's *t*-test. Statistical analysis was performed with Prism v.5.0 (GraphPad Software, La Jolla, CA, USA), and values are expressed as the mean+S.D. of *n* animals.

Values of *P*<0.05 were considered significant.

## Abbreviations

LUT-7G: Luteolin-7-glucoside
IL-22: interleukin 22
IMQ: Imiquimod
