# Supplementary figures and images for: Luteolin-7-glucoside inhibits IL-22/STAT3 pathway, reducing proliferation, acanthosis, and inflammation in keratinocytes and in mouse psoriatic model
Source: Cell Death Dis. 2016 Aug 18;7(8):e2344–. doi: 10.1038/cddis.2016.201 (PMC5108310; doi:10.1038/cddis.2016.201)

Supplementary 1

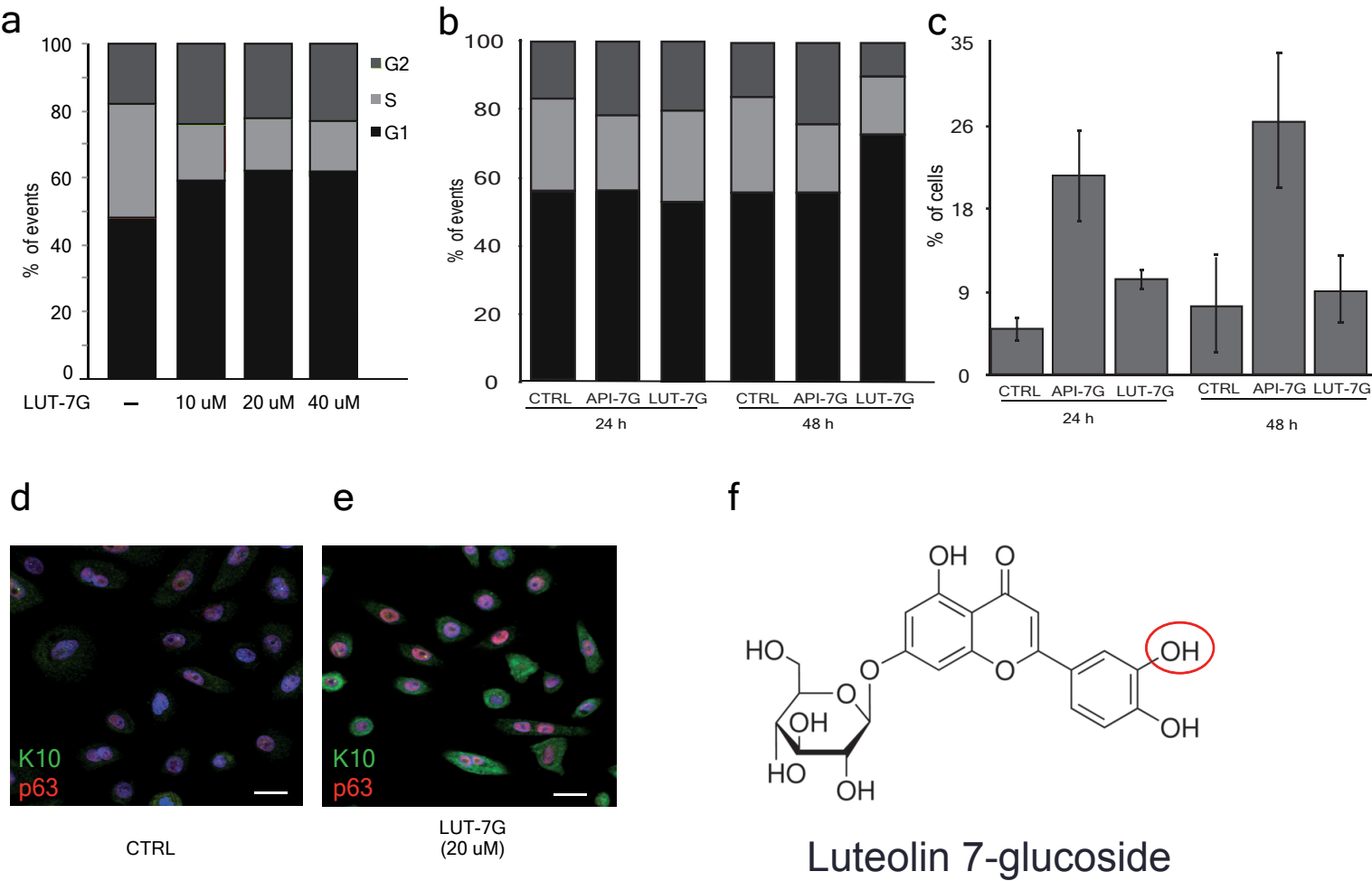

Supplement: Supplementary Figure S1 [file cddis2016201x1.pdf]

## Supplementary 2

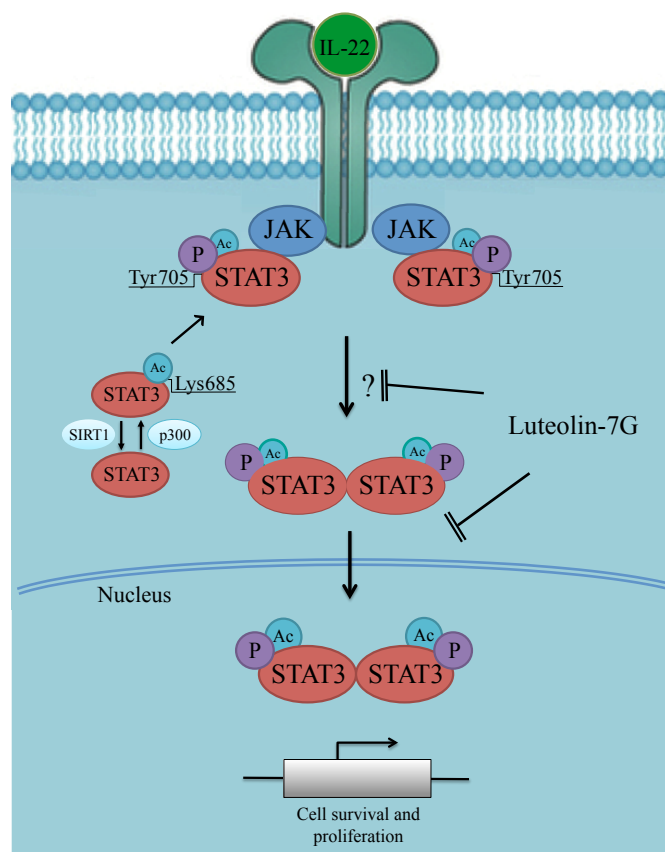

Supplement: Supplementary Figure S2 [file cddis2016201x2.pdf]

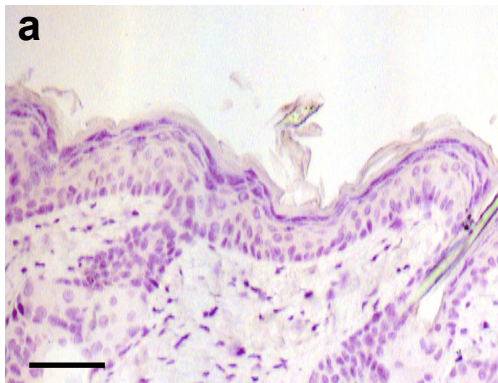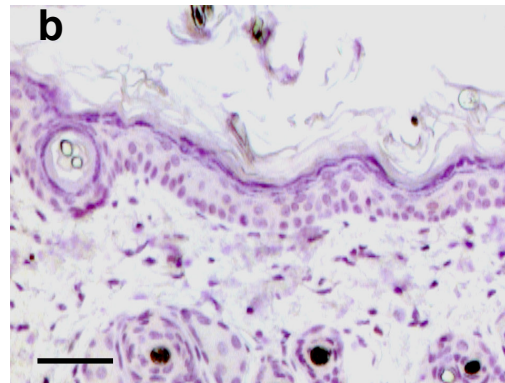

Supplement: Supplementary Figure S3 [file cddis2016201x3.pdf]
