# Supplementary material for: Luteolin-7-glucoside inhibits IL-22/STAT3 pathway, reducing proliferation, acanthosis, and inflammation in keratinocytes and in mouse psoriatic model
Source: Cell Death Dis. 2016 Aug 18;7(8):e2344–. doi: 10.1038/cddis.2016.201 (PMC5108310; doi:10.1038/cddis.2016.201)
Supplement: Supplementary Information [file cddis2016201x4.docx]

**Supplementary Figure Legends**

**Supplementary S1.**

Effects of flavone treatments. To analyse the cell cycle and the apoptotic rate, cells were PI-stained and for each sample 10,000 cells were analysed by cytofluorimetric analysis. (A) P3 Cells were treated with increasing concentration of the flavone Luteolin-7G, to evaluate the effect on the cell cycle progression and to detect the concentration to be used for further investigations. In a range of 10uM until 40 uM, for 3 days, LUT-7G is able to promote an arrest in the G1 phase of the cell cycle. Using both Apigen and Luteolin for HEKn treatment, in their glycosilated form, at a concentration of 20 uM, it was possible to find out API-7G seems to not affect the cell cycle progression (B), like LUT-7G is able to do, after only 48 h of treatment. (C) API-7G can promote the apoptotic process, also after a short time of treatment (24 h), demonstrated by the increased percentage of cells in sub-G1 phase. In contrast LUT-7G does not cause the same activation of apoptotic processes. (D-E) To identify differentiation promoted characteristics (morphological and different expression pattern) P3 cells, after 3 days of LUT-7G treatment and control, were also immunostained with KRT10 and p63 antibodies. Bars (D-E): 25 μm. Molecular structure of LUT-7G, showing the hydroxile group that is lost in apigenin-7-glucoside (F)

**Supplementary S2**

Immunohistochemical analysis of phosphorylated STAT3 in normal and LUT-7G treated mice skin. Analysis using p705Y(Stat3) antibody showed absence of signal in mouse skin treated with LUT-7G alone (B)

Bars (A-B): 40 μm

**Supplementary S3.**

LUT-7G does not allow the translocation into the nucleus of activated STAT3. Cytokines and growth factors induce STAT3 phosphorylation by receptor-associated kinases and then it forms dimers that translocate to the cell nucleus, where they act as transcription activators. STAT3 mediates the expression of a variety of genes that play a key role in many cellular processes such as cell growth, proliferation and apoptosis. The binding of Interleukin 22 to the receptor triggers STAT3 phosphorylation by JAK2. STAT3 phosphorylation is finely regulated also by acetylation of Lys685 residue regulated by p300 acetylates and histone deacetylase SIRT1. Our results demonstrated the inhibition of the translocation of STAT3 into the nucleus, independently from its modification (acetylation and phosphorylation), after luteolin treatment. LUT-7G avoids the dimerization of STAT3 and/or its passage through the nuclear membrane. Thus, STAT3 is not able to activate proliferation gene expression related to psoriasis disease promoted by IL-22 pathway.
